# Sensory Perception of Six Essential Oils in Humans and *Tenebrio molitor*: Relationship with Volatile Compound Physicochemical Properties

**DOI:** 10.3390/molecules31132201

**Published:** 2026-06-23

**Authors:** Antonella Rosa, Alessandra Piras, Silvia Porcedda, Carla Masala, Paolo Solari

**Affiliations:** 1 Department of Biomedical Sciences, University of Cagliari, SP8 Cittadella Universitaria, 09042 Monserrato, CA, Italy; cmasala@unica.it (C.M.); solari@unica.it (P.S.); 2 Department of Chemical and Geological Sciences, University of Cagliari, SP8 Cittadella Universitaria, 09042 Monserrato, CA, Italy; apiras@unica.it (A.P.); porcedda@unica.it (S.P.)

**Keywords:** essential oils, sensory perception, humans, insect, volatiles

## Abstract

Olfactory detection of essential oils (EOs), natural plant-derived mixtures of odorous volatile compounds, stimulates neural pathways involved in emotion, cognitive function, and memory in humans and significantly influences insect behavior (inducing attractiveness or repellency). In this study, the olfactory perception of rose (EO 1, a synthetic mixture with rose aroma), eucalyptus (EO 2), lemon (EO 3), clove (EO 4), rosemary (EO 5), and caraway (EO 6) EOs in untrained human participants was compared to the behavioral responses induced in *Tenebrio molitor* (adult insects) by EO exposure. Significant differences emerged in the perception of EO odor dimensions (pleasantness, intensity, and familiarity) using a Likert-type scale in untrained participants. The tested EOs elicited different behavioral responses in *T. molitor* insects, as assessed by repellency, escape, and choice tests. A positive correlation (r = 0.7861, *p* < 0.05) emerged between EO odor intensity perceived by participants and escape induction in *T. molitor* adults. GC–MS analysis revealed citronellol, 1,8-cineole, limonene, eugenol, α-pinene, and carvone as the most abundant volatile compounds in EO 1, EO 2, EO 3, EO 4, EO 5, and EO 6, respectively. The EO odor dimensions in participants and insect behavioral responses were also related to the in silico physicochemical/pharmacokinetic properties of the main EO components. Our results provide new insights into the chemical basis of olfactory preferences both in *T. molitor* adults and humans.

## 1. Introduction

Essential oils (EOs) are odorous volatile liquids extracted from several parts (flowers, leaves, seeds, peels, roots, branches, bark, wood, gums, or oily resins) of aromatic plants by steam or hydrodistillation, enfleurage, cold pressing, organic solvent extraction, maceration, supercritical fluid extraction, microwave-assisted extraction, and ultrasound-assisted extraction [1,2,3]. EOs are complex mixtures of naturally occurring volatile, aromatic, and lipophilic compounds, including terpenes, terpenoids (oxygenated derivatives such as aldehydes, ethers, ketones, epoxides, and phenols), and phenylpropanoids [3,4,5,6]. EOs are widely used in food, pharmaceutical, and cosmetic industries for their diverse bioactivities, including anti-inflammatory, antioxidant, antimicrobial, antiviral, antidiabetic, analgesic, and anticancer properties [1,2,3,4,5,6,7]. Due to their lipophilic nature and low molecular weights, EO volatile compounds can cross cell membranes, alter phospholipid layers, increase membrane fluidity, and interfere with biological processes at cellular/multicellular levels by interacting with various biological targets [4].

Due to their pleasant aromatic scent and the bioactivity of their compounds, EOs are widely used in “aromatherapy”, a term that collectively refers to their use by oral administration (ingestion), topical application (skin absorption), and inhalation through the nose [1,8,9]. Since ancient times, EOs have been used for both personal fragrance and modification of the living environment [8]. In recent years, they have been increasingly used to improve people’s olfactory environment for possible efficacy in improving mood [8,10]. Administration of natural EOs by inhalation through the olfactory system has been reported to stimulate neural pathways involved in emotion (inducing analgesic, antianxiety, and antistress effects) [2,8], improve cognitive function/memory deficit [11,12], and ameliorate olfactory perception in patients with olfactory deficits using olfactory training [9,13,14]. Several studies have reported the beneficial pharmacological effects of EOs on the central nervous system (CNS) as neuroinflammation modulators in neurodegenerative diseases [2,15,16].

The sense of smell is recognized as one of the most important senses in humans and a key determinant of behavior [17]. Olfactory perception in humans is related to different parameters such as the odorant chemical composition and concentration, the environmental conditions (temperature and humidity) under which the odor is perceived, gender, age, and the subject’s cultural background [8]. In humans, the sensory perception of EO volatile molecules involves a direct route from olfactory receptors (ORs) in the olfactory epithelium to the olfactory bulb, then to the higher olfactory cortex by olfactory sensory neurons, and, notably, to the limbic system (including the amygdala and hippocampus), which is closely related to emotion regulation [8,9,18]. Human sensory perception of EOs is based on odor intensity, familiarity, and hedonic tone (pleasantness), which are strongly linked to the volatility and concentration of specific compounds [8].

EOs are gaining increasing attention as an alternative to synthetic insecticides in the control of insects harmful to crops/stored products for their multiple anti-insect properties (toxic, fumigant, repellent, antifeedant, ovicidal, and larvicidal activities) and favorable eco-toxicological properties, such as low human toxicity, rapid degradation, and low environmental impact (biodegradability) [19,20,21]. Due to their lipophilic nature, EOs can interfere with the basic metabolic, biochemical, physiological, and behavioral functions of insects [19,20,21]. Regarding behavioral effects, EOs can influence insect food consumption, oviposition site choice, and resting site choice by acting as repellents or attractants or by interfering with the recognition of chemical cues. These effects are more pronounced in adults than in immature insects [20,21]. Among insect pests, *Tenebrio molitor* L. (Coleoptera: Tenebrionidae), the yellow mealworm beetle, is dangerous to stored grain and flour in food stores and grocery shops [21,22,23,24]. Considerable efforts have been expended over recent years to use plant-based products like EOs for insect control in stored product pests [21,23]. Several studies have been conducted to evaluate the impact of EOs on the metabolism and behavior of *T. molitor* larvae and adults [21,22,23,24,25].

The *T. molitor* olfactory system is highly specialized for detecting volatiles associated with stored products (grains and cereals), specific pheromones, and chemical signals for mate localization [26,27]. In insects, exogenous lipophilic odorants/volatiles enter the aqueous sensillum lymph through cuticular pores and are subsequently bound and solubilized by odorant-binding proteins (OBPs), a family of small proteins involved in chemoreception, which feature a hydrophobic binding pocket [27,28,29,30]. The OBP–odorant complex is then transported across the sensillum to the specific olfactory receptors (ORs) located on the dendritic membrane, and a transduction cascade is triggered [27,28,29,30]. Several physicochemical characteristics of volatile organic compounds have been demonstrated to drive the sensorial response and attractiveness/repellency of EOs in *T. molitor* [30].

Olfactory perception in insects and humans exhibits remarkable similarities in functional logic and neuroanatomical structure, despite having evolved independently [31]. Both human and insect sensory systems are designed to detect a wide variety of volatile chemicals, and their olfactory preferences are, in part, predetermined by the physicochemical properties of odorant molecules [18,32,33]. The practical application of EOs in protecting stored grain against insect pests is limited by undesirable changes in the sensory characteristics of the treated foodstuff, resulting in products that are not appealing to consumers [34]. Currently, there is growing interest in identifying EOs that combine strong insect-protective properties with favorable sensory attributes [34]. A recent study evaluated the insecticidal activity of various EOs alongside their sensory quality, as assessed by a trained panel of 12 assessors [34]. Moreover, volatile compounds/EOs with pleasant fragrances are desired in cosmetics and are therefore excellent candidates for novel insect repellents [33]. Consequently, the comparative evaluation of human and insect responses to shared EOs, the identification of cross-species convergence in olfactory responsiveness to EOs, and the characterization of physicochemical properties of EO odorant molecules that influence human perception and drive insect responses represent important aspects for the appropriate use of EOs.

**Figure 1:**
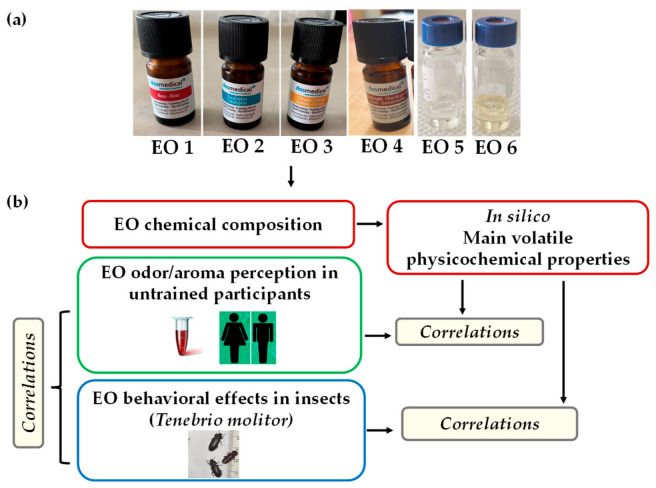
Images of EOs of rose (EO 1, a synthetic mixture with rose aroma), eucalyptus (EO 2), lemon (EO 3), clove (EO 4), rosemary (EO 5), and caraway (EO 6) (**a**) and a schematic workflow diagram of experimental organization (**b**).

In recent studies, we evaluated the differences in the sensory perception of rose (*Rosa damascena*), eucalyptus (*Eucalyptus globulus*), lemon (*Citrus limon*), and clove (*Eugenia caryophyllus*) EOs in non-trained human participants in relation to olfactory function (healthy participants versus participants with hyposmia) [14] and the variation in the sensory perception of rosemary (*Rosmarinus officinalis*) and caraway (*Carum carvi*) EOs in relation to age (young adults and middle-aged participants) [35]. Moreover, the sensory perception of EOs was related to their main volatile components [14,35]. Starting from all these considerations, the present study aimed to extend our previous investigation on these EOs, including rose (EO 1, a synthetic mixture with rose aroma), eucalyptus (EO 2), lemon (EO 3), clove (EO 4), rosemary (EO 5), and caraway (EO 6) (Figure 1a), by comparing their sensory perception in human participants with their sensory perception/behavioral responses in insects. 

*T. molitor* was chosen as an experimental insect model because it has been employed in studies to simulate specific human-related behaviors, particularly in the fields of toxicology, disease, and behavioral ecology [24]. It is considered a highly valued experimental model due to its low cost, rapid life cycle, and easy maintenance under laboratory conditions [22,24]. The olfactory dimensions of EOs, assessed in untrained participants using a hedonic Likert-type scale, were correlated with the behavioral responses of adult *T. molitor* insects to EOs in various behavioral assays. The volatile component profile of EOs was determined by gas chromatography coupled with mass spectrometry (GC–MS). Moreover, EO odor dimensions in humans and the behavioral responses in *T. molitor* were related to the in silico physicochemical properties of the main volatiles to identify common chemical bases underlying odorant detection and behavioral responses. A schematic workflow diagram of the experimental organization of the study is shown in Figure 1b.

The interdisciplinary approach of this study, integrating human sensory evaluation, insect behavioral assays, GC–MS profiling, and in silico physicochemical analyses, links chemical composition to biological perception across two different biological systems. The results provide valuable insights into cross-species sensory convergence and the physicochemical basis of odorant molecules. These findings may contribute to a better understanding of conserved olfactory mechanisms and have potential implications for both sensory science and applied entomology.

## 2. Results

### 2.1. Chemical Composition of EOs

**Figure 2:**
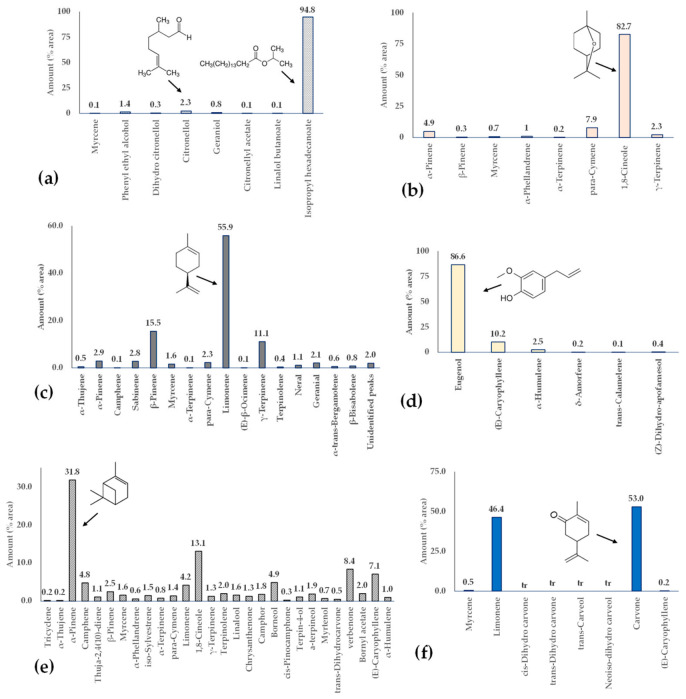
Profile of main volatile compounds of EO 1 (a synthetic mixture with rose aroma) (**a**), EO 2 (eucalyptus) (**b**), EO 3 (lemon) (**c**), EO 4 (clove) (**d**), EO 5 (rosemary) (**e**), and EO 6 (caraway) obtained by GC–MS analysis (**f**). Semi-quantitative analysis was performed by peak area normalization (expressed as % peak area) [14,35]. The chemical structure of the most abundant volatiles was also reported.

The chemical composition (expressed as % area) of EOs from rose (EO 1), eucalyptus (EO 2), lemon (EO 3), clove (EO 4), rosemary (EO 5), and caraway (EO 6), obtained by GC/MS analysis, is reported in Figure 2. Rose EO 1 was characterized by a high amount of isopropyl hexadecanoate (94.8% of total components), an odorless organic ester used as a diluent, due to the high price of rose EO (Figure 2a). Citronellol (2.3%), phenyl ethyl alcohol (1.4%), and geraniol (0.8%) were identified as the main EO 1 volatile components, followed by small amounts of dihydro citronellol (0.3%), citronellyl acetate (0.1%), linalol butanoate (0.1%), and myrcene (0.1%). The high amount of diluent qualified EO 1 as a synthetic rather than a genuine plant EO (a synthetic mixture with rose aroma).

The most abundant volatile compounds identified in EO 2 (eucalyptus) were 1,8-cineole (monoterpene cyclic ether, 82.7%) and para-cymene (monoterpene hydrocarbon, 7.9%), followed by lower amounts of α-pinene (4.9%), γ-terpinene (2.3%), and α-phellandrene (1%) (Figure 2b).

Limonene was found to be the major component in EO 3 (lemon), accounting for 55.9%, followed by β-pinene (15.5%) and γ-terpinene (11.1%). Other components, with relatively small amounts, were α-pinene (2.9%), sabinene (2.8%), para-cymene (2.3%), geranial (2.1%), and myrcene (1.6%) (Figure 2c).

GC–MS analysis allowed measurement of high amounts of the phenylpropanoid eugenol (86.6%) in EO 4 (clove), followed by (*E*)-caryophyllene (10.2%) and α-humulene (2.5%), and very small amounts of δ-amorfene, *trans*-calamelene, and (*Z*)-dihydro-apofarnesol (Figure 2d).

Among the 28 compounds identified in EO 5, α-pinene represented the major component (31.8%), followed by 1,8-cineole (13.1%), verbenone (8.4%), (E)-caryophyllene (7.1%), borneol (4.9%), camphene (4.8%), and limonene (4.2%) (Figure 2e). Small amounts of β-pinene (2.5%), terpinolene (2.0%), and bornyl acetate (2.0%) were also found.

The most abundant volatile compounds found in EO 6 were carvone (53.0%) and limonene (46.4%), whereas myrcene (0.5%) and (E)-caryophyllene (0.2%) were present in small amounts (Figure 2f).

### 2.2. Sensory Perception of EOs in Untrained Participants

Two populations of untrained participants were enrolled to assess the sensory properties of EOs 1–4 [14] and EOs 5–6 [35].

Table 1 shows the demographic and clinical features of the participants in the EO sensory analysis, including age, sex, weight, height, and body mass index (BMI). Values of odor threshold (OThr), odor discrimination (ODi), odor identification (OId), and TDI score (OThr + ODi + OId scores), indicating the olfactory function of the participants, are also reported in Table 1. 

No statistically significant differences emerged between the two different groups in age, weight, height, BMI, and olfactory function (OThr, Odi, OId, and TDI). Therefore, the results of the sensory perception of the six EOs obtained from previous independent cohorts were combined in this study [14,35].

**Figure 3:**
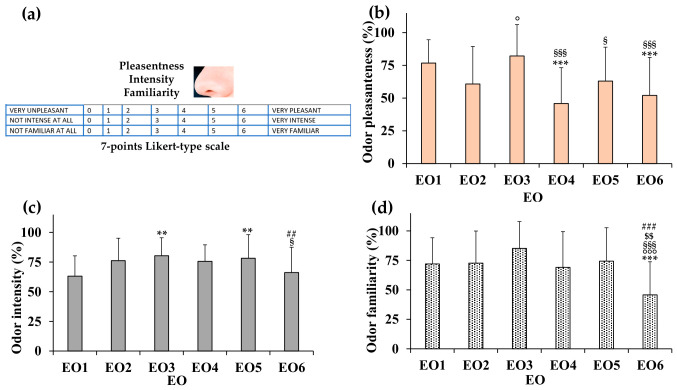
Hedonic Likert-type scale (7-points, from 0 to 6) used for the assessment of olfactory perception of EO 1 (a synthetic mixture with rose aroma), EO 2 (eucalyptus), EO 3 (lemon), EO 4 (clove), EO 5 (rosemary), and caraway (EO 6) (**a**) and % values (considering point 6 as 100% of perception) of odor pleasantness (**b**), intensity (**c**), and familiarity (**d**) of EOs 1–6 [14,35]. Data are presented as mean values and standard deviations. Significant differences between groups (one-way ANOVA adjusted with the Bonferroni multiple comparisons test): *** = *p* < 0.001, ** = *p* < 0.01 versus EO 1; °°° = *p* < 0.001, ° = *p* < 0.05 versus EO 2; ^§§§^ = *p* < 0.001, ^§^ = *p* < 0.05 versus EO 3; ^$$^ = *p* < 0.01 versus EO 4; ^###^ = *p* < 0.001, ^##^ = *p* < 0.01 versus EO 5.

A seven-point hedonic Likert-type scale (from 0 to 6) (Figure 3a) was used to evaluate odor pleasantness (OP), intensity (OI), and familiarity (OF) of EO 1 (rose), EO 2 (eucalyptus), EO 3 (lemon), EO 4 (clove), rosemary (EO 5), and caraway (EO 6) in untrained participants to evidence differences in the sensory perception of various EOs.

Percentual values of OP, OI, and OF (calculated considering point 6 as 100%) determined for EOs 1–6 are reported in Figure 3b–d. Significant differences were observed in %OP (Figure 3b), %OI (Figure 3c), and %OF (Figure 3d) among EOs 1–6. In general, the EO 3 odor emerged as the most pleasant (EO 3 > EO 1 > EO 5 > EO 2 > EO 6 > EO 4), intense (EO 3 > EO 5 > EO 2 > EO 4 > EO 6 > EO 1), and familiar (EO 3 > EO 5 > EO 2 > EO 1 > EO 4 > EO 6). Instead, EO 4, EO 1, and EO 6 odors were perceived as the least pleasant, intense, and familiar, respectively.

Subjective descriptions of the odor (aroma) of the six EOs furnished by untrained participants are listed in Table S1. Descriptors of the odor of EOs (The Good Scents Company) [36,37,38,39,40,41] and their main constituents (PubChem) [42] are also reported in Table S1.

The specific odors (aroma) of rose, eucalyptus, lemon, clove, and rosemary were recognized by several participants for EO 1, EO 2, EO 3, EO 4, and EO 5, respectively, while none of them identified the odor of caraway.

Despite the presence of a high quantity of isopropyl hexadecanoate (odorless diluent), the floral/rose scent was identified by participants, essentially attributable to the main EO 1 components, including citronellol (fresh rosy odor) and phenyl ethyl alcohol (rose-like odor) [42].

The herbal, eucalyptus, camphoreous, and medicinal aroma of EO 2 was identified by several untrained participants, attributable to the odor characteristics of its main components, including 1,8-cineole (camphor-like), para-cymene (sweetish aromatic), and α-pinene (turpentine, pine) [42].

EO 3 (lemon) odor was primarily associated with a citrus odor type, influenced by aromatic terpenes like limonene (citrus-like) [42].

Sensory descriptors used by participants for EO 4 (cloves, incense, cinnamon, spices, herbs, eucalyptus, natural plant) were similar to those reported in the literature (spicy, aromatic, balsamic, woody, and minty) [39], attributable to its main components, like eugenol (cloves, warm, spicy, floral) and *(E)*-caryophyllene (woody-spicy, dry, and clove-like) [42].

Regarding EO 5, some participants recognized rosemary EO, probably due to the high concentration of its main components α-pinene (turpentine, pine) and 1,8-cineole (camphor-like) [42].

The odor of EO 6 (caraway) was not identified by participants, but odor-perceived attributes furnished by participants were similar to those reported in the literature for this EO (herbal, camphoraceous, woody, aromatic, minty, balsamic, and medicinal) [41].

### 2.3. Assessment of EO Detectability in T. molitor Insects (Behavioral Tests)

**Figure 4:**
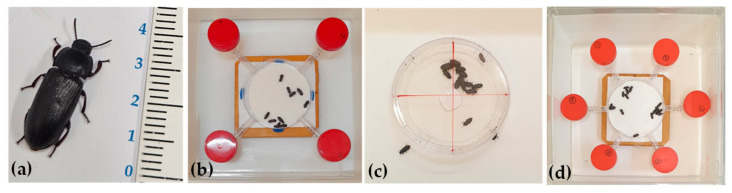
Image of an adult of *T. molitor* (**a**). Experimental arenas used in the repellence (closed four-exit arena) (**b**), escape (open four-exit arena) (**c**), and choice (closed six-exit arena) (**d**) assays for the sensory evaluation/behavioral tests of EO 1 (a synthetic mixture with rose aroma), EO 2 (eucalyptus), EO 3 (lemon), EO 4 (clove), EO 5 (rosemary), and caraway (EO 6) in *T. molitor* adults.

The behavior (repellence or attractiveness) of adult individuals of *T. molitor* in response to the sensory perception of EOs 1–6 was assessed using three different experimental arenas (Figure 4).

**Figure 5:**
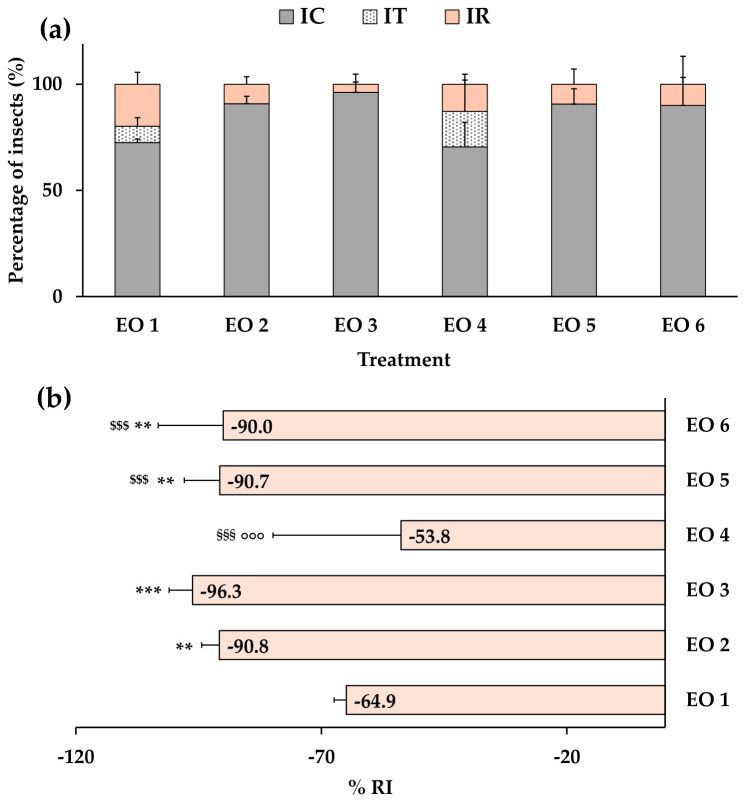
Percentage of insects that remained in the center of the closed four-exit arena (%IR), insects that entered in the control box (water, %IC), and those that entered in the treatment (%IT) (**a**), and values of % repellency index (%RI) (**b**) determined for EO 1 (a synthetic mixture with rose aroma), EO 2 (eucalyptus), EO 3 (lemon), EO 4 (clove), EO 5 (rosemary), and EO 6 (caraway) (repellence assay) after 24 h of treatment. Data are presented as the mean values and standard deviations of six independent experiments (n = 6), using 20 adult insects per replicate. Significant differences between groups (one-way ANOVA adjusted with the Bonferroni multiple comparisons test): *** = *p* < 0.001, ** = *p* < 0.01 versus EO 1; °°° = *p* < 0.001 versus EO 2; ^§§§^ = *p* < 0.001 versus EO 3; ^$$$^ = *p* < 0.01 versus EO 4.

In the first experimental series, conducted in the closed four-exit arena with external containers (repellency assay), the analysis considered the percentage distribution of insects among two control containers (C), two treatment containers (T), and the arena (R). Figure 5 shows the percentage of insects that remained in the center of the closed four-exit arena (%IR), insects that entered in the control (water, %IC), and those that entered in the treatment (%IT) (Figure 5a), and % values of repellency index (%RI) (Figure 5b) determined for EOs 1–6 after 24 h of exposure (repellency assay).

High %IC values were observed for lemon EO 3 (96.3%), eucalyptus EO 2 (90.8%), rosemary EO 5 (90.7%), and caraway EO 6 (90.0%), whereas rose EO 1 (72.6%) and clove EO 4 (70.5%) showed lower percentages.

In all trials, the proportion of individuals recorded in the control containers was higher than that observed in the containers associated with EOs. Regarding insects found in the treatment, %IT values of 16.7% and 7.6% were determined for EO 4 and EO 1, respectively. Instead, no insects were found after 24 h of exposure in boxes containing the other EOs.

Negative values of %RI were calculated for all tested EOs, following the order: lemon EO 3 (−96.3%) > eucalyptus EO 2 (−90.8%) ≈ rosemary EO 5 (−90.7%) ≈ caraway EO 6 (−90.0%) > rose EO 1 (−64.9%) > clove EO 4 (−53.8%). All EOs emerged as repellents for insects; clove and rose EOs displayed significantly lower repellency versus *T. molitor* adults than the other tested EOs.

**Figure 6:**
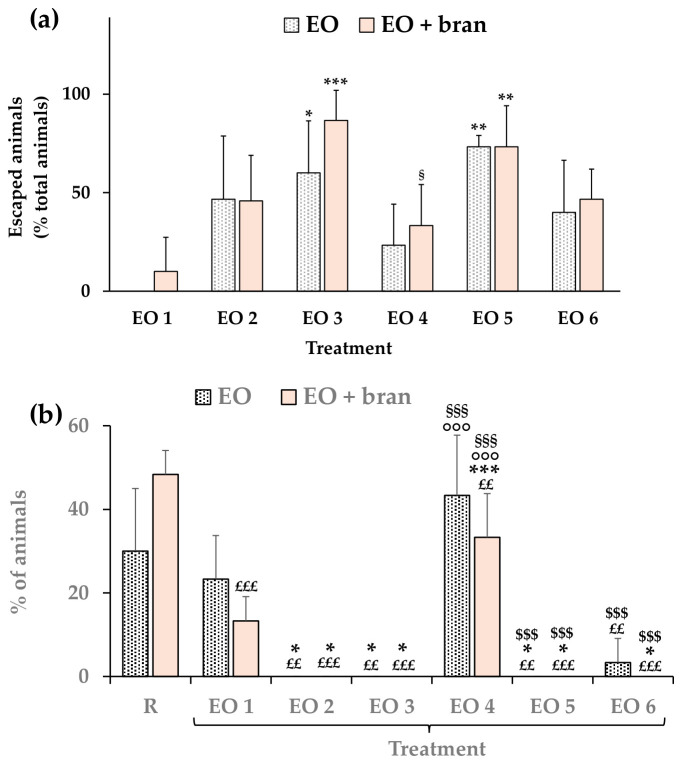
Percentage of animals that escaped from the open four-exit arena (escape assay) after 10 min in the presence of EO 1 (a synthetic mixture with rose aroma), EO 2 (eucalyptus), EO 3 (lemon), EO 4 (clove), EO 5 (rosemary), and EO 6 (caraway) presented, alone or dispersed in the bran, in the center of the arena (**a**). Percentage of animals that remained in the center of the closed six-exit arena (R) and animals that entered in the external boxes containing different EOs 1–6 (%Choice) presented alone or dispersed in the bran (choice assay) after 24 h of treatment (**b**). Data are presented as the mean values and standard deviations of six independent experiments (n = 6) using 10 (escape assay) or 20 (choice assay) adult insects per replicate. Significant differences between groups (one-way ANOVA adjusted with the Bonferroni multiple comparisons test): ^£££^ = *p* < 0.001, ^££^ = *p* < 0.01 versus R; *** = *p* < 0.001, ** = *p* < 0.01, * = *p* < 0.05 versus EO 1; °°° = *p* < 0.001 versus EO 2; ^§§§^ = *p* < 0.001, ^§^ = *p* < 0.05 versus EO 3; ^$$$^ = *p* < 0.01 versus EO 4.

The tendency of insects to escape in the presence of EOs 1–6 was then assessed using an open four-exit arena without external containers (escape assay) over a short exposure time (10 min). Each EO, pure or dispersed in the bran, was put in the center of the arena in the presence of all insects. The percentage of animals that escaped from the arena in the presence of each EO or EO + bran was calculated with respect to the total number of insects (escaped from arena + remained in arena). As reported in Figure 6a, in trials with each EO alone, the % of escaped insects, reflecting the repellent strength of the EOs, followed the order: rosemary EO 5 (73.3%) > lemon EO 3 (60.0%) > eucalyptus EO 2 (46.7%) > caraway EO 6 (40.0%) > clove EO 4 (23.3%) > rose EO 1 (0.0%).

A similar trend was observed in the presence of each EO dispersed in the bran, and the percentages of escaped insects (or %Escape) were 86.7%, 73.3%, 46.7%, 45.8%, 33.3%, and 10.0% for lemon, rosemary, caraway, eucalyptus, clove, and rose EOs, respectively. Overall, the addition of bran did not appear to attenuate the repellent effect of the different EOs.

Finally, the ability of *T. molitor* adults to select among EOs 1–6, contemporaneously presented in six external boxes, was assessed using a closed six-exit arena (choice assay). Figure 6b shows the % values of insects remaining, after 24 h of treatment, in the center of the arena (residual, R) and % of insects that entered in the external boxes containing each different EO (%Choice), with respect to the total number of insects initially released into the arena. EOs 1–6 were presented as pure EO or dispersed in the bran (EO + bran). When presented as pure EOs, insects chose to move preferentially toward clove EO 4 (43.3% of total insects), rose EO 1 (23.3%), and caraway EO 6 (3.3%). Insects did not enter the external boxes containing eucalyptus, lemon, and rosemary EOs, reflecting the high repellent strength observed in the repellency and escape assays.

Table S2 depicts the literature data on the bioactivity of the tested EOs for *T. molitor* (adult insects and larvae) and other insect species [43,44,45,46,47,48,49,50,51,52,53,54,55]. Our data confirmed the repellent effect of all EOs, as previously reported, with a different degree of potency.

### 2.4. Relation Between EO Sensory Perception in Untrained Participants and EO Detectability in T. molitor Insects (Behavioral Tests)

**Figure 7:**
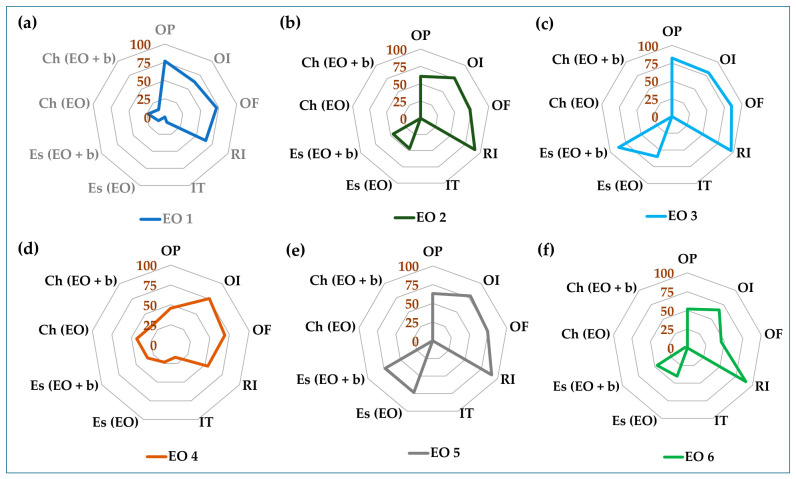
Bioactivity radars of EO 1 (a synthetic mixture with rose aroma) (**a**), EO 2 (eucalyptus) (**b**), EO 3 (lemon) (**c**), EO 4 (clove) (**d**), EO 5 (rosemary) (**e**), and EO 6 (caraway) (**f**) computed considering odor dimensions measured in untrained participants (% values of OP, OI, and OF), % values of RI and IT obtained from repellency assay, % values of escape in the presence of EO and EO + bran (escape assay, Es), and % values of choice in the presence of EO and EO + bran (choice assay, Ch).

To provide an overall comparison of the results obtained with the EOs across human and insect models, the bioactivity radars (Figure 7) were computed for each EO considering human odor dimensions (% values of OP, OI, and OF), %RI and %IT (repellency assay), % values of escape in the presence of EO or EO + bran (escape assay), and % values of choice in the presence of EO or EO + bran (choice assay).

This graphical representation highlighted similarities/differences between various EOs regarding their total bioactivity profile. Bioactivity radars of lemon EO 3 (Figure 7c) and rosemary EO 5 (Figure 7e) were very similar, evidencing high values of repellency/escape induction and high ratings in human perception. Eucalyptus EO 2 (Figure 7b) and caraway EO 6 (Figure 7f) showed bioactivity radars close enough to EO 3 and EO 5, with some differences in the induction of escape behavior in insects. Bioactivity radars of rose EO 1 (Figure 7a) and clove EO 4 (Figure 7d) were different from those of the other EOs for their lower capacity to induce repellency and escape behavior.

**Figure 8:**
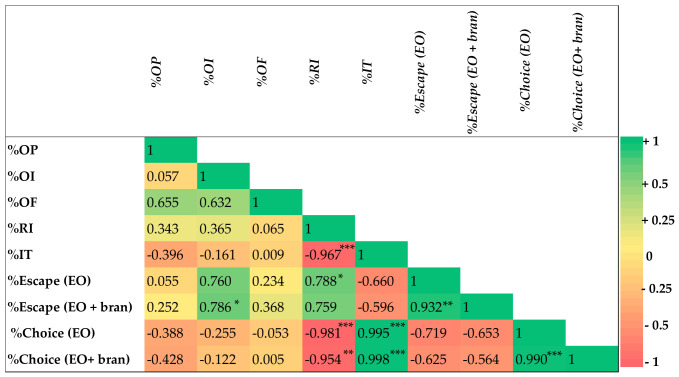
Heatmap of Pearson’s correlations (r) and significance (*** = *p* < 0.001, ** = *p* < 0.01; * = *p* < 0.05) calculated for EO odor dimension values measured in untrained participants (%OP, %OI, and %OF) versus scores obtained for EOs in *T. molitor* adults, including %RI and %IT (from repellency assay), % escape in the presence of EO or EO + bran (escape assay), and % choice in the presence of EO or EO + bran (choice assay).

Then, we tried to identify potential relations between EO sensory perception in untrained participants and EO detectability in *T. molitor* insects as obtained by behavioral tests. Pearson’s correlations (r) were determined for EOs 1–6 between values of odor dimensions and results obtained by insect behavioral assays, as reported in the bioactivity radars. The heatmap of obtained Pearson coefficient (r) values and significance is reported in Figure 8.

In the heatmap analysis, red indicates a strong negative correlation, and green indicates a strong positive correlation.

For sensory dimensions of EOs 1–6 obtained in untrained participants, slight positive correlations were found between %OP/%OF (r = 0.6548) and %OI/%OF (r = 0.6321), indicating that familiarity with an odor stimulus could increase its perceived pleasantness and intensity.

Regarding insect behavior models, positive correlations were determined between %RI/%Escape (EO) (r = 0.7884, *p* < 0.05) and %RI/%Escape (EO + bran) (r = 0.7591), evidencing a similar action of tested EOs in two different experimental models (insect repellency and escape induction) and thus validating the observed insect behaviors. Negative correlations emerged between %RI/%Choice (EO) (r = −0.9814, *p* < 0.001) and %RI/%Choice (EO + bran) (r = −0.9537, *p* < 0.01), highlighting the capacity of insects to move versus the less repellent EO. The ability of insects to distinguish between different EOs (when presented alone and in groups) was also confirmed by the linear positive relation observed between the value of %IT, obtained in the closed four-exit arena closed, and values obtained in the closed six-exit arena (%IT/%Choice (EO), r = 0.9951, *p* < 0.001; %RI/%Choice (EO + bran) (r = 0.9985, *p* < 0.001).

Regarding potential relations between EO sensory perception in untrained participants and insects, from the heatmap correlation matrix, a positive correlation was determined between %OI/%Escape (EO) (r = 0.7602) and %OI/%Escape (EO + bran) (r = 0.7861, *p* < 0.05), evidencing the potential role of EO intensity in the escape induction (at a short time of exposure) in *T. molitor* adults.

### 2.5. Correlation Between EO Sensory Perception in Untrained Participants and Insects and Physicochemical and Pharmacokinetic Properties of EO Main Volatile Components

The canonical SMILES (Simplified Molecular-Input Line Entry Specification nomenclature) (Table S3), molecular weight (MW), rotatable bond count (RBC), hydrogen bond donor count (HBDC), hydrogen bond acceptor count (HBAC), lipophilicity (XLogP3-AA), complexity, topological polar surface area (TPSA), and vapor pressure (VP) of the main volatile compounds of EOs 1–6, including citronellol for EO 1, 1,8-cineole for EO 2, limonene for EO 3, eugenol for EO4, α-pinene for EO 5, and carvone for EO 6 (Table S4), were obtained from the free web database PubChem [42]. Other important in silico pharmacokinetic properties, such as lipophilicity (Consensus Log P_o/w_), water solubility (Log S, as the mean value of ESOL, Ali, and SILICOS-IT), human gastrointestinal absorption (HIA), skin permeation (Log Kp), blood–brain barrier (BBB) permeability, and central nervous system (CNS) permeability (Table S4), were obtained by entering the canonical SMILES of selected compounds into the freely accessible web tools SwissADME [56] and pkCSM-pharmacokinetics [57].

Finally, to identify the principal EO characteristics affecting sensory perception/behavioral responses in participants/insects, Pearson’s correlations (r) were determined between several computed properties of citronellol, 1,8-cineole, limonene, eugenol, α-pinene, and carvone and their parameters assessed in humans and *T. molitor* assays.

**Figure 9:**
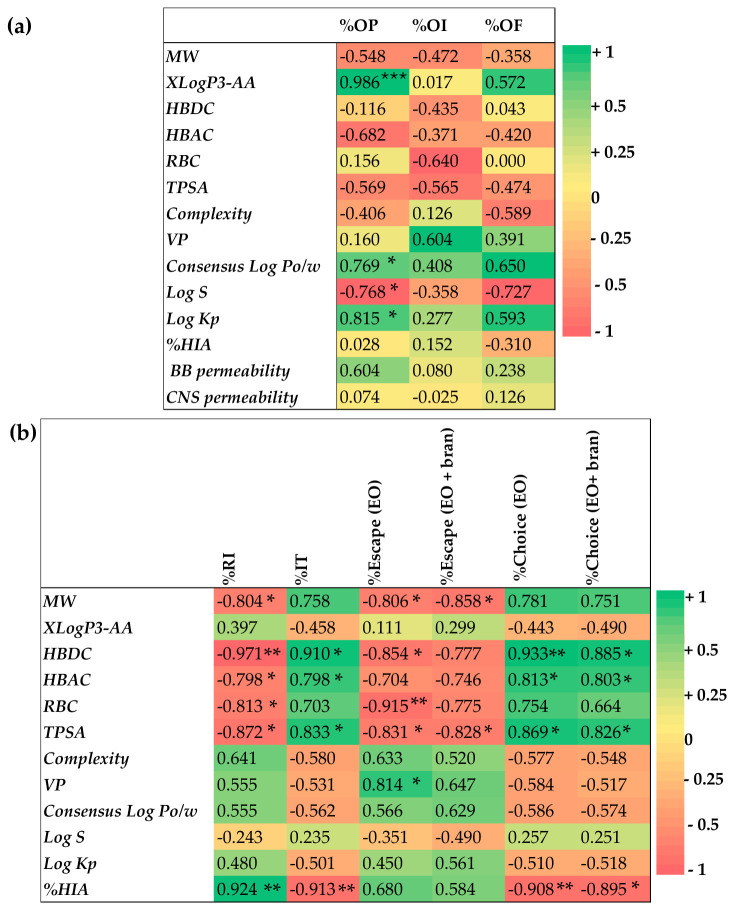
Heatmap of Pearson’s correlations (r) and significance (*** = *p* < 0.001, ** = *p* < 0.01; * = *p* < 0.05) calculated between a series of selected computed properties of EO main volatiles reported in Table S4 and EO odor dimension values measured in untrained participants (%OP, %OI, and %OF) (**a**) or scores obtained for EOs in *T. molitor* adults, including %RI and %IT (repellency assay), %Escape in the presence of EO or EO + bran (escape assay), and %Choice in the presence of EO or EO + bran (choice assay) (**b**).

Figure 9a shows the heatmap of Pearson coefficients (r) and significance calculated between EO sensory dimensions in the participants (%OP, %OI, and %OF) and computed properties of EO main volatiles reported in Table S4, including MW, HBDC, HBAC, RBC, TPSA, complexity, VP, lipophilicity (XLogP3-AA and Consensus Log P_o/w_), Log S, Log Kp, %HIA, BBB permeability, and CNS permeability.

Positive correlations were measured between %OP/XLogP3-AA (r = 0.986, *p* < 0.001), %OP/Consensus Log P_o/w_ (r = 0.769, *p* < 0.05), and %OP/Log Kp (r = 0.815, *p* < 0.05), while %OP emerged as negatively correlated to Log S (r = −0.768, *p* < 0.05), evidencing a potential role of compound lipophilicity and capacity to permeate epithelial cells in affecting EO odor pleasantness. A positive association emerged between the volatility values of EO main components and the intensity of the EO olfactory perception (%OI/VP, r = 0.604). Odor familiarity of EOs showed similar correlations, unless weaker, of pleasantness.

Figure 9b shows the heatmap of r values calculated between scores obtained for EOs in *T. molitor* adults, such as %RI and %IT (from repellency assay), %Escape in the presence of EO or EO + bran (escape assay), and %Choice in the presence of EO or EO + bran (choice assay), and computed properties of EO main volatiles reported in Table S4, including MW, HBDC, HBAC, RBC, TPSA, complexity, VP, lipophilicity (XLogP3-AA and Consensus Log P_o/w_), Log S, Log Kp, and HIA.

EO repellency (%RI) and capacity to induce escape, including %Escape (EO) and %Escape (EO + bran), emerged as negatively correlated to several physicochemical properties of the main volatiles, such as MW, HBDC, HBAC, RBC, and TPSA. Interestingly, positive associations emerged between the EO repellent effect and the bioavailability (ability to cross biological membranes) of the main components (%RI/%HIA, r = 0.924, *p* < 0.01) and between the EOs’ ability to induce escape and the main component volatility (%Escape/VP, r = 0.814, *p* < 0.05). Conversely, strong positive correlations were observed between %IT, %Choice (EO), and %Choice (EO + bran) and MW, HBDC, HBAC, RBC, and TPSA. Strong negative correlations were found for %IT/%HIA (r = −0.913, *p* < 0.01), %Choice (EO)/%HIA (r = −0.908, *p* < 0.01) and %Choice (EO + bran)/%HIA (r = −0.895, *p* < 0.05). These correlations suggested that less bioavailable volatiles were likely less repellent.

## 3. Discussion

EOs are complex natural mixtures of volatile, lipophilic, and odoriferous substances obtained from aromatic plants. They are constituted by a blend of 20 to 70 organic compounds, some of which represent more than 80% of the constituents [21]. Generally, the main components are responsible for the activity of EO; however, in some instances, it has been demonstrated that all EO constituents work synergistically to produce the biological effect [2,6,21]. Since ancient times, EOs have been widely used worldwide for their diverse biological activities to treat a variety of illnesses, preserve food from deterioration, and regulate emotions (as functional fragrances) [1,8,32,58]. Nowadays, their use is continuously growing due to the strong demand for pure natural ingredients for pharmaceutical, flavor/fragrance, cosmetic, aromatherapy, phytomedicine, food/beverage, and household industries [58]. EOs have also been proposed as low-environmental-impact alternatives for controlling insect pests harmful to crops and human health [20].

The practical application of EOs for protecting stored grain against insect pests is limited by undesirable changes in the sensory characteristics of treated food [34]. There is increasing interest in identifying EOs that combine favorable sensory properties for humans with strong protective effects against insects [34]. A previous study evaluated the chemical composition, olfactory profile (intensity, pleasantness, and persistence) in trained assessors, and toxicity against the stored grain pest *Sitophilus granaries* of various EOs (*Foeniculum vulgare*, *Pistacia lentiscus*, and *Ocimum basilicum*) [34]. Moreover, another study matched the smell profiles of *Artemisia verlotiorum*, *Lavandula dentata* L., and *Ruta chalepensis* EOs, assessed by a trained panel composed of 13 assessors, with their repellence, oviposition deterrence, and larvicidal activity against the mosquito *Aedes albopictus* (Skuse) for their possible use as ingredients in topical repellents [59].

In this study, we provide a comparative analysis of olfactory perception of selected EOs (rose, eucalyptus, lemon, clove, rosemary, and caraway EOs) in humans and behavioral responses elicited by the same EOs in *T. molitor* adults, integrating human sensory evaluation, insect behavioral assays, EO chemical profiling, and in silico physicochemical/pharmacokinetic analysis of the EOs’ most abundant volatile constituents. This study, directly correlating human odor dimensions with insect olfactory-driven behavior using the same EO panel, offers new insights into conserved chemical mechanisms underlying olfactory-driven preferences across taxa. The identification of cross-species sensory convergence and the physicochemical determinants of odorant perception in both humans and insects has potential implications for advancing sensory science and applied entomology [30,34].

The chemical profiles of EOs 1–6 were assessed by GC–MS analysis [14,35]. Commercial rose EO 1 was characterized by isopropyl hexadecanoate (isopropyl palmitate) as the predominant compound. This ester is an odorless organic compound used for dilution of the rose EO due to its high production cost [14]. Therefore, EO 1 is an oil with a nearly fully synthetic composition, rather than a genuine plant EO (a synthetic mixture with rose aroma). Excluding isopropyl hexadecanoate, EO 1 emerged as rich in citronellol, followed by phenyl ethyl alcohol and geraniol. 1,8-cineole (or eucalyptol), a monoterpene cyclic ether, was identified as the most abundant volatile compound in eucalyptus EO 2, followed by para-cymene. Limonene, followed by β-pinene and γ-terpinene, were the most abundant components in lemon EO 3. High amounts of the terpen eugenol, followed by lower amounts of (*E*)-caryophyllene and α-humulene, were measured in clove EO 4. α-Pinene emerged as the major component, followed by 1,8-cineole and verbenone in rosemary EO 5. Carvone represented the most abundant volatile in caraway EO 6; however, a high quantity of limonene was also measured. EOs are complex mixtures of volatiles; however, two or three components are usually present in large proportions (20–70%) [2]. Our findings on composition were perfectly in line with previous literature data that indicate citronellol, 1,8-cineole, limonene, eugenol, α-pinene, and carvone as the major compounds found in EOs of *R. damascena* flowers [60], *E. globulus* leaves [4,22,45,46,61], *C. limon* peel [48,62], *E. caryophyllus* stem [22,50,61], *R. officinalis* leaves [52], and *C. carvi* seeds [22,54,63], respectively.

Then, the odor dimensions of EOs 1–6 were assessed and compared in untrained participants using a seven-point labeled hedonic Likert-type scale, a method previously used for the evaluation of the odor perception of aromatized food products [64,65]. The sensory evaluation revealed marked differences among EOs in perceived pleasantness, intensity, and familiarity. Lemon EO 3 odor emerged as the most pleasant, intense, and familiar odor. This finding is consistent with previous literature reporting citrus odors as generally well tolerated and positively evaluated by participants [8], likely due to cultural exposure and frequent use in food, hygiene, and household products. Clove (EO 4) and caraway (EO 6) were rated as less pleasant and familiar among EOs. The odor of these EOs is dominated by the phenylpropanoid eugenol and the ketone carvone, respectively, compounds often associated with intense and pungent/spicy or medicinal notes [42,66,67] that may elicit mixed hedonic responses in assessors. The spicy/pungent odor of eugenol and carvone is attributable to their ability to stimulate the trigeminal nerve [64,65]. EO 1 was defined as the least intense by participants due to its high dilution rate with the odorless excipient; however, the rose scent of EO 1 was still recognized. The persistence of recognizable odor qualities in rose EO 1, despite its dilution, highlights the low olfactory thresholds and high perceptual impact of citronellol (fresh rosy/rose odor), phenyl ethyl alcohol (rose-like), and geraniol (sweet rose, floral, and geranium odor) [42].

Considering the subjective description of odor (aroma), several untrained participants recognized rose, eucalyptus, lemon, clove, and rosemary EOs. In general, the subjective descriptors were very similar to those reported on a specialized website (The Good Scents Company Information System) [36,37,38,39,40,41].

Importantly, both the pleasantness and perceived intensity of EOs 1–6 were positively correlated with familiarity, supporting the notion that previous exposure and cognitive recognition modulate the hedonic evaluation of odors. Our data are consistent with a previous study on odor perception and the emotion-regulating functions of EOs, which reported a positive correlation between odor pleasantness and familiarity and a weak correlation between subjective odor intensity and emotional perception [8].

The correlational analysis conducted between the EOs 1–6 olfactory dimensions and the main in silico physicochemical and pharmacokinetic properties of the EOs’ main volatile compounds (citronellol for EO 1, 1,8-cineole for EO 2, limonene for EO 3, eugenol for EO4, α-pinene for EO 5, and carvone for EO 6) evidenced the role of the chemical composition of the tested EOs in the modulation of their sensory perception in the participants. Odor pleasantness correlated positively with lipophilicity (XLogP3-AA and Consensus Log P_o/w_) and epithelial permeation capacity (Log Kp) and negatively with water solubility (Log S), suggesting that compound ability to cross biological membranes, related to lipophilicity, may generate more pleasant olfactory experiences. Moreover, a positive correlation was determined between the vapor pressure (VP) of the main components and the EO odor intensity, evidencing the potential role of EO compound volatility in the EO olfactory perception [33,66]. Our data evidenced that the human olfactory perception of EOs is related to their chemical composition and the physicochemical properties of their main volatile components, as previously reported [8,32,68].

Olfactory perception of an odorant stimulus in humans is related to characteristics of the perceiver (gender, age, cultural background) and odorant (chemical composition, concentration, and physical properties) [8,18,32]. In humans, odorant recognition is mediated by a great repertoire of olfactory receptors (ORs), G-protein-coupled receptors located across the plasma membranes of the ciliated dendrites of olfactory sensory neurons (OSNs), localized in the nasal cavity’s olfactory epithelium (covered by the mucus) [8,9,32]. The inhalation of the EOs’ volatile molecules stimulates the olfactory pathway, which principally involves the binding of odorant molecules to ORs and the activation of the olfactory nerve, extending from the nose toward the brain [8,9,18]. Hydrophobic and volatile odorant molecules need to be transported through the aqueous mucus by the small soluble proteins, the odorant-binding proteins (OBPs), to cross the hydrophilic barrier of the mucus and bind to ORs [32]. In the olfactory pathway, transcellular, paracellular, and intracellular pathways, through the olfactory nerve and olfactory bulb, represent the modes of EO cell-to-cell diffusion [66]. Molecular size, volatility, hydrophobicity, and the presence of specific functional groups have been previously identified as important chemical and physical properties of volatile/odorant molecules that influence their interaction with ORs/OBPs and determine the perceived odor [18,32,68]. A previous study evidenced that the chemical type significantly influences the odor sensory evaluation of a series of EOs [8]. Some EO molecules can pass through the olfactory mucosa (olfactory pathways), depending on their molecular sizes [68]. The EO penetration through the olfactory nerve, which is connected to brain areas, allows the generation of several cellular and molecular events (regulation of neuronal pathways) [68].

Then, the behavioral responses of *T. molitor* to EOs 1–6 were assessed. All tested EOs exhibited repellent activity toward *T. molitor* adults, albeit with significantly different intensities. Lemon EO 3, eucalyptus EO 2, rosemary EO 5, and caraway EO 6 showed high values of repellency and escape-inducing effects towards *T. molitor* adults across assays. Clove EO 4 and rose EO 1 displayed significantly lower repellency/escape induction versus *T. molitor* adults than the other EOs. When EOs 1–6 were presented together (choice assay), insects chose to move preferentially toward clove EO 4 and rose EO 1, reflecting their lower repellent strength with respect to the other EOs.

Literature data evidenced high fumigant and contact toxicity in adult *Tribolium castaneum* [43] and contact, repellent, and ovicidal effects on *Tetranychus urticae* [44] of *R. damascena* EO. Repellent effects and mortality induction on *Tribolium confusum*, *T. molitor*, and *Acanthoscelides obtectus* adults and potent fumigant toxicity against rice weevil adults (*Sitophilus oryzae*) were previously reported for eucalyptus EO [23,45]. Repellent (>89.0% repellence index at 12 h), fumigant, and contact activity against both *T. molitor* larvae and adults [47], strong fumigant toxicity against *Sitophilus oryzae* adults [48], and mortality for *Plodia interpunctella* eggs [49] were previously reported for lemon peel EO. Previous studies evidenced the toxic and repellent effect of clove EO on *Sitophilus granarius* adults [50] and its capacity to induce mortality and repellency on *T. molitor* in larva, pupa, and adult stages [51]. Rosemary EO 5 showed potent fumigant properties/toxicity against *S. oryzae* [45,49] and *Tyrophagus putrescentiae* [49], contact toxicity against *T. putrescentiae* and *Trichomyrmex destructor* [48,52], and significant repellent activity against *T. destructor* [53]. High contact toxicity against *S. oryzae*, *Rhizopertha dominica*, and *T. castaneum* adults [48,55] and fumigant toxicity against *T. molitor* and *T. confusum* adults [54] were previously reported for caraway EO. Previous studies evidenced the insecticidal effects of citronellol [44], 1,8-cineole [45], limonene [47], eugenol [24,49,50,51], α-pinene [69], and carvone [22,54], major volatile compounds found in EO 1, EO 2, EO 3, EO 4, EO 5, and EO 6, respectively.

The convergence of results obtained from the repellency, escape, and choice assays supports the robustness of the observed behavioral patterns and validates the use of multi-arena testing to characterize insect sensory-driven responses. The incapacity of insects to enter arenas containing highly repellent eucalyptus EO 2, lemon EO 3, and rosemary EO 5, dominated by limonene, 1,8-cineole, and α-pinene, respectively, further supports the strong deterrent properties of monoterpene-rich oils [23,45]. Interestingly, rose EO 1 and clove EO 4, although repellent in isolated contexts, showed comparatively higher attractiveness in the choice assay when all six EOs were presented simultaneously. This suggests that insect behavioral decisions are context-dependent and influenced by relative odor contrasts rather than absolute repellent strength alone. Such results are consistent with ecological scenarios in which insects weigh multiple olfactory cues before selecting or avoiding a substrate [20].

The correlational analysis conducted between behavioral responses obtained in *T. molitor* assays and selected in silico physicochemical and pharmacokinetic properties of the EOs 1–6 main volatile compounds evidenced that repellency and escape behaviors were positively correlated with predicted human intestinal absorption (%HIA, measure of bioavailability of a compound) and VP, respectively. These correlations indicated that highly volatile and bioavailable (able to penetrate biological membranes) compounds are more readily detected and trigger stronger avoidance responses in *T. molitor* insects. Conversely, the positive correlations that occurred between physicochemical properties, such as MW, HBDC, HBAC, RBC, and TPSA, and choice behavior evidenced that heavier, more polar molecules with lower permeability/bioavailability were associated with reduced repellent effects and increased choice behavior. This evidenced that less hydrophobic and less bioavailable volatiles were probably less repellent.

Plant volatiles are important factors that affect the ability of insects to locate host plants, and their value is mainly reflected as an attractant and repellent [70,71]. Volatile chemicals that repel insects typically act on the olfactory system [71]. The principal olfactory organs in insects are the antennae and maxillary palps, organs covered with hair-like projections called sensilla that house a small number of olfactory receptor neurons (ORNs) [71]. The sensillar sheath is perforated with pores that allow compounds to enter into contact with the inner lymph bathing the neurons [71]. The *T. molitor* olfactory system is highly specialized for detecting volatile organic compounds associated with food sources, stored products, specific pheromones, and chemical signals for mate localization [26,27,28,29,30]. In *T. molitor*, volatile hydrophobic compounds (odorants) are typically transported in aqueous media by small soluble carrier proteins (odorant-binding proteins, OBPs), contained in the lymph within the antennal sensilla [27,28,29,30]. OBPs (approximately 12–14 kDa in *T. molitor*) are responsible for volatile organic compound solubilization, transportation, and delivery to receptors (ORs) on the sensory dendrites [27,28]. OBPs, characterized by an internal hydrophobic binding pocket, are necessary for safeguarding exogenous hydrophobic volatiles against degradation before their interaction with the corresponding ORs [28]. The conformation of OBPs’ hydrophobic binding pocket depends on both the insect species and the structures of the odorant compounds [29]. Studies on insects/*T. molitor* olfaction consistently showed that sensory perception is driven by small volatile hydrophobic compounds that share specific structural and physicochemical properties, which determine whether the molecule can adequately evaporate to reach the antennae, bind to OBPs, and be transported to activate ORs [27,28,29,30]. OBPs, as ligand transporters, are critical for odor detection and therefore represent potential targets for the discovery of new repellent compounds that may modulate olfactory responses [29]. Chemical–physical characteristics of the volatile compounds strictly affect the interaction with insect OBPs and ORs [29,30,33].

The results obtained in the behavioral tests evidenced that the efficacy of EOs as repellents against *T. molitor* adults was directly related to the physicochemical properties of their major constituents, mainly volatility and computed bioavailability/ability to cross biological membranes. High volatility of an EO organic component promotes its rapid evaporation and facility to reach the antennae. Conversely, the ability to cross the membrane, which is related to lipophilicity, influences the capacity to interact with OBPs or penetrate insect cuticles by dissolving in body surface lipids. Hydrophobic EOs cause water stress in insects by blocking the spiracles of their exoskeletons, resulting in suffocation and pain in cuticular waxes [30]. Our findings align with current models of insect chemoreception, in which OBPs selectively transport small, hydrophobic molecules toward ORs. Therefore, an increase in the polarity of volatiles is likely due to reduced access to ORs within sensillar structures. The increase in the hydrophilicity/polar character of a volatile ligand could increase the affinity/binding to OBPs, which can lead to a diminished effect on insects, acting as a form of “sequestration resistance”. In this mechanism, the OBPs, which usually transport odors to ORs, instead trap, bind, or neutralize compounds before they can trigger a negative, toxic, or attractive response [70,71].

Both humans and *T. molitor* possess sensory systems capable of detecting a wide variety of volatile chemicals, with perceptual dimensions of odor, such as intensity and hedonic valence (pleasant/unpleasant), being conserved across species [31]. Olfactory preferences both in insects and mammals are, in part, predetermined by the physicochemical properties of odorant molecules [18,32,33]. One of the most relevant findings of this study is the positive correlation between odor intensity perceived by humans and escape behavior induced in *T. molitor* adults. This relationship suggests that odor salience, driven primarily by volatility and bioavailability of odorant molecules, could play a central role in behavioral avoidance across both humans and insects. Despite substantial differences in olfactory receptor repertoires and neural organization between insects and mammals, both systems rely on physicochemical features of odorant molecules for detection and signal transduction. The observed correlation supports the hypothesis that certain fundamental chemical properties of volatiles, such as VP and lipophilicity, create a common framework influencing olfactory-driven behavior independently of species-specific receptor architecture. Notably, no direct relationship emerged between human odor pleasantness and insect repellency, reinforcing that hedonic valuation in humans and behavioral avoidance in insects reflect distinct evolutionary pressures. In humans, hedonic perception is shaped by prior experience, associative learning, and emotional context, whereas in insects, avoidance behavior is more closely associated with evolutionary pressures related to ecological fitness, particularly the detection and avoidance of potentially toxic stimuli.

The limitations of this study are the use of two independent cohorts rather than a single unified panel to evaluate human sensory perception of EOs 1–6 and the use of only six essential oils for the assessment of Pearson correlations between sensory variables, insect responses, and molecular descriptors, with a consequent instability of correlation coefficients. Additionally, only one major volatile per EO was selected for physicochemical correlation, whereas synergistic or antagonistic interactions among minor components may also influence perception and behavior. Future studies should explore concentration-dependent effects and test broader EO panels. At the mechanistic level, coupling behavioral assays with electrophysiological recordings and molecular docking studies on insect OBPs and receptors could further clarify structure–activity relationships.

## 4. Materials and Methods

### 4.1. Tested EOs 1–6

EOs of rose (*R. damascena* flower extract diluted in isopropyl hexadecanoate, EO 1), eucalyptus (*E. globulus* leaf/twig oil, EO 2), lemon (*C. limon* peel oil, EO 3), and clove (*E. caryophyllus* stem oil, EO 4) (lot n. SO-29082022-01 of StimuScent Dos Medical smell training kit) [14] were purchased from Dos Medical (Sense Trading BV, Groningen, The Netherlands). The tested rosemary EO (EO 5) was supplied by the company Erbe Matte (Sant’Antioco, SU, Italy), lot n. 03/2015 [35]. *C. carvi* EO (EO 6) was obtained by supercritical extraction from the seeds of the plant harvested in Lithuania as previously described [72].

### 4.2. GC–MS Analysis of EOs

EO analysis was carried out by gas chromatography/mass spectrometry (GC–MS), using a gas chromatograph (Agilent 7820A, Agilent Technologies, Santa Clara, CA, USA) coupled with a mass selective detector having an electron ionization device (EI) and a quadrupole analyzer (Agilent 5975, Agilent Technologies, Santa Clara, CA, USA), as previously reported [14,35]. The Agilent MSD ChemStation software (rev. E.01.00.237) was used to handle and analyze chromatograms and mass spectra. Compounds were identified by comparing their experimental retention indices and mass spectra with those reported in the literature and library spectra [73,74]. Retention indices for the components were calculated using the retention times of two standard n-alkane mixes (C_8_–C_20_ and C_21_–C_40_) [75]. Semi-quantitative analysis was performed by peak area normalization as previously reported [14,76].

### 4.3. Assessment of EO Olfactory Perception in Untrained Participants

#### 4.3.1. Participants

Two populations of untrained individuals were enrolled to assess the sensory perception of EOs: 30 participants for EOs 1–4 [14] and 59 participants for EOs 5–6 [35]. The two participant cohorts had different sample sizes because they were derived from two previous studies conducted sequentially [14,35]. All participants involved in this study received an explanatory statement and signed a written informed consent. Demographic features (age, weight, height, and body mass index) were collected for all participants. Both groups had similar demographic characteristics and were tested under identical experimental conditions, including the same inclusion and exclusion criteria, evaluators, and methodologies. Inclusion criteria included age ≥ 18 years, good general health, and the ability to understand and perform the test. Exclusion criteria included respiratory infections, head or neck trauma, neurodegenerative diseases, stroke, head/neck injury, cancer or chemotherapy, diabetes, pregnancy, severe cognitive impairment, and asthma [14,35]. The participants did not take any medications for 5 days before the test. All participants were evaluated individually in a well-ventilated room under controlled conditions. Data collection was performed from November 2024 to December 2025 according to the Declaration of Helsinki. The Ethical Committee of the University of Cagliari (Protocol number: 3605, 1 October 2024) approved this study.

#### 4.3.2. Assessment of Olfactory Function

The assessment of olfactory function was performed with the Sniffin’ Sticks test (Burghart Messtechnik, Wedel, Germany), a validated tool for routine clinical assessment of olfactory function in subjects [14,35,64,65]. This test consists of pen-like odor-dispensing devices for the evaluation of odor threshold (OThr), odor discrimination (ODi), and identification (OId) as previously reported [64,65]. Not all participants were allowed to smoke or use scented products before the chemosensory evaluation. The OThr scores were assessed using 16 stepwise dilutions of n-butanol and ranged from 16 (perception of the lowest n-butanol concentration) to 1 (no perception of the highest concentration). For the ODi test, three different pens were used, two containing the same odor and the third containing the odor target, and the ODi scores ranged from 0 to 16 (sum of correct answers). The OId test was assessed by 16 common odors as previously reported. The sum of OThr + ODi + OId represented the total olfactory score (TDI).

#### 4.3.3. Assessment of EO Odor Dimensions (Pleasantness, Intensity, and Familiarity)

Before the sensory assessment, each EO was aliquoted at room temperature (23 °C) in 2 mL glass test bottles. All participants were instructed to drink only water 1 h before the experiment and not to wear any scented products on the day of testing. EOs were tested for the olfactory analyses in a randomized order and without any dilution. The exposure time for each EO was 3–4 s. The washout interval between each EO presentation was 20–30 s. The odor pleasantness (OP), intensity (OI), and familiarity (OF) of the EOs were evaluated using a self-reported 7-point Likert-type scale (a hedonic scale method) (Figure 3a), which ranged from 0—not at all to 6 (such as 0 = very unpleasant and 6 = very pleasant; 0 = not intense at all and 6 = very intense; 0 = not familiar at all and 6 = very familiar) [64,65]. A value of 3 was considered a neutral point. The labeled hedonic Likert scale is amply used in food science to measure hedonic differences among beverages, foods, and consumer products and predict their acceptance, suitable for use without extensive training [62,63]. EO odor dimensions were expressed as % values, calculated considering point 6 of the Likert-type scale as 100%. Moreover, the subjects generated subjective odor (aroma) attributes/descriptors perceived with more intensity. The complete session test, including olfactory function and EO odor rating assessment, was around 1 h. All assessments were carried out at room temperature (23 °C) in a ventilated room during the daytime (9 a.m. to 6 p.m.).

### 4.4. Assessment of EO Detectability in T. molitor Insects (Behavioral Tests)

#### 4.4.1. Insects and Rearing Conditions

Adults of *T. molitor* used for the experimental trials were obtained from a colony reared in the insectary of the Department of Biomedical Sciences, Section of Physiology, University of Cagliari (Cagliari, Italy). The species, easily reared under laboratory conditions, was maintained in open plastic boxes containing a layer of soft wheat bran as the rearing substrate. The boxes were organized according to the month of rearing, allowing an approximate estimation of the age of the individuals. Insects were kept at a constant temperature of 25 °C and a relative humidity of 60% in 12 h light/12 h dark photoperiod. Fresh fruit pieces (particularly apples and bananas) were periodically provided in each container for insect feeding and hydration.

#### 4.4.2. Repellency Assay

Twenty adult individuals of *T. molitor* (Figure 4a) (age of about 1 month, approximately 1:1 male-to-female ratio) were placed in the center of a closed four-exit arena positioned inside a transparent plastic container (Figure 4b). The arena consisted of a large Petri dish (diameter of 14 cm) with four holes, each connected via a plastic tunnel (6.5 cm) to an external small, closed plastic box. A disk of absorbent paper was placed on the bottom of the dish. Two boxes contained water (control) presented in thin, cotton discs, whereas the other two boxes contained thin cotton discs, each impregnated with 25 μL of a single pure EO under investigation. Treatment and control containers were arranged diagonally. The arena was closed with a transparent, perforated top. Before testing, the insects were not fed for 24 h preceding the experiments. In this repellency test, after the acclimation phase (10 min), insects were allowed to move freely for 24 h within the experimental arena or move toward one of the four available containers. After 24 h of treatment, the number of insects found in the control containers (CI), in the treatment containers (TI), and those remaining in the arena (RI) were determined with respect to the total number of insects used (TotI). Then, the percentual repellency index (%RI) was calculated according to the following formula: %RI = [(TI − CI)/TotI] × 100. Positive %RI values indicated attraction towards the treatment, whereas negative values indicated repellency. During the experiments, the environmental conditions were 25 °C, 60% relative humidity, and 12 h light/12 h dark photoperiodic regime. Experiments were carried out in an air-conditioned room with a continuous air exchange system for odor removal to guarantee cleaning procedures between trials and avoid odor carryover. Among consecutive trials, the arena was rotated to change the positions of treatments and controls and reduce potential effects caused by local differences in experimental conditions (lighting or environmental disturbances).

#### 4.4.3. Escape Assay

Ten adult individuals of *T. molitor* (age of about 1 month, approximately 1:1 male-to-female ratio) were placed in the center of an open four-exit arena positioned inside a transparent plastic container. The arena consisted of a Petri dish (as described in Section 4.4.2) with four holes (exits) that directly led to the external container (Figure 4c). During the experiments, the arena was closed with a transparent top. In this escape test, after the acclimation phase (5 min), the insects were exposed for 10 min to each EO (25 μL) presented in thin cotton discs, as pure EO or dispersed in the bran, at the center of the arena. Control trials using bran alone, without EOs, were also performed. Before testing, the insects were not fed for 24 h preceding the experiments. During the experiment, the insects were free to move within the arena or to leave it through one of the available exits. For each trial, data were analyzed calculating, after exposure, the percentage of insects remaining in the arena (residual) and the percentage of those that moved outside the arena (% Escape) with respect to the total number of insects initially released into the arena at the start of the study. The experiments were carried out in an air-conditioned room with a continuous air exchange system for odor removal; the environmental conditions were 25 °C, 60% relative humidity, and exposure to light.

#### 4.4.4. Choice Assay

Twenty adult individuals (age of about 1 month, approximately 1:1 male-to-female ratio) of *T. molitor* were placed in the center of a closed six-exit arena positioned inside a transparent plastic container (Figure 4d). The six-exit arena differed from the four-exit arena in the presence of six holes in the Petri dish, connected via plastic tunnels to six external closed plastic boxes. Each box contained one of EOs 1–6 (25 μL) presented, in thin cotton discs, as pure EO or dispersed in the bran. In this choice assay, EOs 1–6 were tested simultaneously. The arena was closed with a transparent, perforated top. Before testing, the insects were not fed for 24 h preceding the experiments. After the acclimation phase (10 min), the insects were allowed to move freely for 24 h within the experimental area or move toward one of the six boxes containing EOs 1–6. The experiments were carried out in an air-conditioned room with a continuous air exchange system for odor removal. During the tests, the environmental conditions were 25 °C, 60% relative humidity, and 12 h light/12 h dark photoperiodic regime. In subsequent trials, the arena was rotated to modify the spatial arrangement of treatments. After 24 h of exposure, data were analyzed by calculating the percentage of insects remaining in the arena (residual) and the percentages of insects that moved into each box containing different EOs (%Choice), with respect to the total number of insects initially released into the arena at the start of the study.

### 4.5. In Silico Evaluation of Physicochemical and Pharmacokinetic Properties of the Main Volatile Components of EOs

Physicochemical properties (MW, XLogP3-AA, HBDC, HBAC, RBC, TPSA, and complexity) and simplified molecular-input line entry specification (canonical SMILES) nomenclature of the main identified volatile components of EOs, including citronellol for EO 1, 1,8-cineole for EO 2, limonene for EO 3, eugenol for EO 4, α-pinene for EO 5, and carvone for EO 6, were obtained from the free web database PubChem [42]. The canonical SMILES of the compounds were introduced into the freely accessible web tools SwissADME [56] and pkCSM-pharmacokinetics [57]. The SwissADME web tool provided pharmacokinetics parameters of compounds, including lipophilicity (Consensus Log P_o/w_), water solubility (Log S), gastrointestinal absorption (according to the white of the BOILED-Egg model), and BBB permeation (according to the yolk of the BOILED-Egg model) [14,56]. The pkCSM-pharmacokinetics furnished several pharmacokinetics/toxicity properties of compounds, including intestinal absorption (%HIA), the logarithm of the ratio of compound concentration in the brain and the blood (log BB), and the logarithm of blood–brain permeability-surface area product (log PS) [14,57].

### 4.6. Statistical Analyses

For the human model, an a priori power analysis was conducted using G*Power 3.1 to estimate the required sample size. Based on previous studies [14,35,64] using similar protocols, the analysis indicated a total required sample size of 42 participants (21 for each group) to detect a large effect size (Cohen’s d = 0.80) with an alpha level of 0.05 and a statistical power of 0.80. For the insect assays, data were presented as means and standard deviations of six independent experiments (n = 6), with 10 (escape assay) or 20 (repellency and choice assays) adult insects per replicate. Six independent replicates (n = 6) are widely considered adequate for behavioral insect studies under controlled laboratory conditions.

Initially, the D’Agostino & Pearson test was used to assess the normality of the data distribution using the software package SPSS software version 25 for Windows (IBM, Armonk, NY, USA). GraphPad Prism version 10.0.0 for Windows (GraphPad Software, Boston, MA, USA) was used to estimate the statistical differences between different data groups. Student’s unpaired *t*-test with Welch’s correction (populations are not assumed to have the same standard deviation) was used to compare the means of the two groups. One-way analysis of variance (one-way ANOVA), followed by the Bonferroni multiple comparisons test, was used to perform multiple comparisons of the group means. The minimal level of significance was *p* < 0.05. Bivariate correlations using Pearson’s coefficient (r) were applied to evaluate potential correlations between EO odor dimensions (%OP, %OI, and %OF) obtained in untrained participants and EO detectability (%RI, %IT, %Escape, and %Choice) in *T. molitor* behavioral assays. Pearson correlations were also determined between several computed properties of citronellol, 1,8-cineole, limonene, eugenol, α-pinene, and carvone, reported in Table S4, and EO sensory dimensions/behavioural responses of EOs 1–6 in humans and insects. The correlation analysis was used to evaluate the strength and direction of the linear relationship between two quantitative variables, resulting in a correlation coefficient that ranged from −1 to +1.

## 5. Conclusions

Overall, this study demonstrates that fundamental physicochemical properties of EOs shape olfactory perception and olfactory-driven behavior in both humans and insects. The shared dependence on volatility, lipophilicity, and bioavailability highlights conserved chemical determinants of odor detection despite evolutionary divergence. These findings contribute to a deeper understanding of olfaction across species and may inform the rational design of EO-based products for aromatherapy, environmental modulation, and eco-friendly insect control strategies.

## Figures and Tables

**Table 1:** Demographic and clinical features of participants in EO sensory analysis.

Parameters	EO 1–4 (n = 30) [14]	EO 5–6 (n = 59) [35]
Age	40.6 ± 16.3	33.9 ± 15.0
Sex	16 W/14 M	39 W/20M
Weight (kg)	63.0 ± 10.0	62.7 ± 11.1
Height (m)	1.6 ± 0.1	1.6 ± 0.1
BMI	23.5 ± 3.4	23.2 ± 3.6
OThr	8.1 ± 3.4	9.4 ± 4.5
ODi	12.5 ± 1.9	12.4 ± 2.1
OId	12.8 ± 1.4	12.8 ± 2.0
TDI score	32.5 ± 6.2	34.5 ± 6.3

Legend: BMI = body mass index; OThr = odor threshold; ODi = odor discrimination; OId = odor identification; TDI score = OThr + ODi + OId scores. Data are presented as mean values and standard deviations. No significant differences emerged between groups (Student’s unpaired *t*-test with Welch’s correction).

## Data Availability

The datasets generated and analyzed during the current study are available from the corresponding author on reasonable request.

## Supplementary Materials

The following supporting information can be downloaded at: https://www.mdpi.com/article/10.3390/molecules31132201/s1, Table S1: Odor (aroma) perceived attributes furnished by untrained participants and odor descriptors obtained from the literature of EOs 1–6; Table S2: Effects in insects of EOs 1–6 from literature data; Table S3: Canonical smiles of citronellol, 1,8-cineole, limonene, eugenol, α-pinene, and carvone; Table S4: Physicochemical and pharmacokinetic properties of citronellol, 1,8-cineole, limonene, eugenol, α-pinene, and carvone.
