# Supplementary material for: Sensory Perception of Six Essential Oils in Humans and Tenebrio molitor: Relationship with Volatile Compound Physicochemical Properties
Source: Molecules. 2026 Jun 23;31(13):2201. doi: 10.3390/molecules31132201 (PMC13363658; doi:10.3390/molecules31132201)
Supplement: Supplementary file 1 [file molecules-31-02201-s001.zip › molecules-4354929-supplementary.pdf]

Article

Sensory Perception of Six Essential Oils in Humans and *Tenebrio molitor*: Relationship with Volatile Compound Physicochemical Properties

Antonella Rosa [1,*], Alessandra Piras [2], Silvia Porcedda [2], Carla Masala [1,†], and Paolo Solari [1,†]

[1] Department of Biomedical Sciences, University of Cagliari, SP8 Cittadella Universitaria, 09042 Monserrato, CA, Italy; anrosa@unica.it (A.R.); cmasala@unica.it (C.M.); solari@unica.it (P.S.);
[2] Department of Chemical and Geological Sciences, University of Cagliari, SP 8 Cittadella Universitaria, 09042 Monserrato, CA, Italy; apiras@unica.it (A.P.); porcedda@unica.it (S.P.)
[*] Correspondence: anrosa@unica.it
[†] These authors contributed equally to this work.

Table of Contents

Table S1: Odor (aroma) perceived attributes furnished by untrained participants and odor descriptors obtained from literature of EOs 1-6.

Table S2: Effects in insects of EOs 1-6 from literature data.

Table S3: Canonical smiles of citronellol, 1,8-cineole, limonene, eugenol, α-pinene, and carvone.

Table S4: Physicochemical and pharmacokinetic properties of citronellol, 1,8-cineole, limonene, eugenol, α-pinene, and carvone.

Table S1 Odor (aroma) perceived attributes furnished by untrained participants and odor descriptors obtained from literature of EO 1 (a synthetic 1mixture with rose aroma), EO 2 (eucalyptus), EO 3 (lemon), EO 4 (clove), EO 5 (rosemary), and EO 6 (*C. carvi*). Odor descriptors obtained from PubChem [41] for EOs main components (MC), identified by GC-MS, are also reported.

EO	EO perceived odor in participants	EO human perception	Main components (MC)	Odor in human of MC [42]
EO 1	Rose, pungent, pleasant, vanilla, citrus fruits, marseille soap, violet, flowers, scots pine, fir, citronella, natural essence, honey, spice.	Odor type: floral.<br>Odor description: floral, rose, spicy, winey, waxy, honey, green, metallic, artichoke [36].	[1]Isopropyl hexadecanoate<br>[2]Citronellol<br>Phenyl ethyl alcohol<br>Geraniol	Odorless<br>Fresh rosy, rose<br>Rose-like odor<br>Sweet rose, floral, geranium-like
EO 2	Eucalyptus, fennel, licorice, anise, herbs, mint, menthol, balsamic plants, lavender, camphor, tiger balm, tea tree oil, peppermint, unpleasant, aromatic, medicine.	Odor type: herbal.<br>Odor description: herbal, eucalyptus, camphoreous, medicinal [37].	[2]1,8-Cineole<br>para-Cymene<br>α-Pinene<br>γ-Terpinene	Camphor-like<br>Sweetish aromatic, weak citrus]<br>Turpentine, pine<br>Herbaceous-citrusy
EO 3	Lemon, Citrus, orange, lemon and orange, mandarin, ginger, yuck, tea, clementine, sweet, fresh.	Odor type: citrus.<br>Odor description: citrus, lemon, lemon peel [38].	[2]Limonene<br>β-Pinene<br>γ-Terpinene<br>para-Cymene<br>α-Pinene<br>Sabinene<br>Geranial	Citrus-like<br>Turpentine, dry, woody, resinous<br>Herbaceous-citrusy<br>Sweetish aromatic, weak citrus<br>Turpentine, pine<br>Warm, oily-peppery, woody-herbaceous<br>Lemon-like, strong lemon
EO 4	Cloves, incense, cinnamon, mild cinnamon, smoked, spices, herbs, leather, eucalyptus, lavender, natural plant, unpleasant.	Odor Type: spicy.<br>Odor description: spicy, aromatic, balsamic, woody, fruity, minty, phenolic powdery [39].	[2]Eugenol<br>*(E)*-Caryophyllene<br>α-Humulene	Cloves, warm, spicy, floral<br>Woody-spicy, dry, clove-like aroma<br>Woody, oceanic-watery, spicy-clove
EO 5	Rosemary, eucalyptus, Citrus, bergamot, balsamic, lavender-like, helichrysum, aromatic plant, herbs, bark, menthol, scots pine, mint, saffron-like, turpentine.	Odor Type: herbal.<br>Odor description: herbal, camphoreous, woody, aromatic, minty, balsamic, medicinal, phenolic [40].	[2]α-Pinene<br>1,8-Cineole<br>Verbenone<br>*(E)*-Caryophyllene<br>Borneol<br>Camphene<br>Limonene	Turpentine, pine<br>Camphor-like<br>Minty spicy<br>Woody-spicy, dry, clove-like aroma<br>Sharp camphor-like<br>Camphor-like<br>Citrus-like
EO 6	Aromatic plant, herbs, mint. forest scent, anise, fennel, spice, vanilla, artichoke, unpleasant, menthol, pleasant, licorice.	Odor Type: herbal.<br>Odor description: fresh, herbal, spicy, minty, balsamic, bread rye, bread seedy, carrot, carvone [41].	[2]Carvone<br>Limonene	Caraway, bread rye<br>Citrus-like

[1]Odorless organic compound used as a diluent. [2]Most abundant volatile compound.

Table S2 Effects in insects of EO 1 (a synthetic mixture with rose aroma), EO 2 (eucalyptus), EO 3 (lemon), EO 4 (clove), EO 5 (rosemary), and EO 6 (*C. carvi*) from literature data.

**EO**	**EO effects in insects**
EO 1	High fumigant and contact toxicity in adult *Tribolium castaneum* and low repulsive effects against larval stage of *T. castaneum* [43].<br>Contact, repellent and ovicidal effects on the different life stages of *Tetranychus urticae* [44].
EO 2	Low repellent activity on *T. molitor* larvae [22].<br>Repellent effects on *Tribolium confusum*, *T. molitor*, and *Acanthoscelides obtectus* adults; noticeable mortality on the *T. confusum* and *A. objects* adults, moderately low mortality in *T. molitor* adults [23].<br>Potent fumigant toxicity against rice weevil adults (*Sitophilus oryzae*) [45].<br>Weak or very limited repellent effect on *T. molitor* larvae [46].
EO 3	Repellent (>89.0% repellence index at 12 h), fumigant, and contact activity against both *T. molitor* larvae and adults [47].<br>Strong fumigant toxicity against *Sitophilus oryzae* adults [48].<br>Mortality for *Plodia interpunctella* eggs, [49].
EO 4	Moderate repellent activity for *T. molitor* larvae [22].<br>Toxic and repellent effect to *Sitophilus granarius* adults [50].<br>Mortality and repellency on *T. molitor* in larva, pupa, and adult stages [51].
EO 5	Potent fumigant toxicity against rice weevil adults (*Sitophilus oryzae*) [45].<br>Strong fumigant properties against *Sitophilus oryzae* [49].<br>Fumigant and contact toxicity against *Tyrophagus putrescentiae* [49].<br>Significant insecticidal activity against *Agrotis ipsilon* larvae [52].<br>Contact toxicity and repellent activity against *Trichomyrmex destructor* [53].
EO 6	High contact toxicity against *Sitophilus oryzae* adults [48].<br>Fumigant toxicity against *T. molitor* and *T. confusum* adults [54].<br>Contact toxicity against *S. oryzae*, *Rhizopertha dominica*, *T. castaneum* adults [54].

[1]Odorless organic compound used as a diluent. [2]Most abundant volatile compound.

Table S3. Canonical smiles, obtained by PubChem web database [42], of the main identified volatile components of EO 1-6, including citronellol for EO 1 1,8-cineole for EO 2, limonene for EO 3, eugenol for EO 4, α-pinene for EO 5, and carvone for EO 6.

Compound name	[1]Canonical SMILES
Citronellol	CC(CCC=C(C)C)CCO
1,8-Cineole	CC1(C2CCC(O1)(CC2)C)C
Limonene	CC1=CCC(CC1)C(=C)C
Eugenol	COC1=C(C=CC(=C1)CC=C)O
α-Pinene	CC1=CCC2CC1C2(C)C
Carvone	CC1=CCC(CC1=O)C(=C)C

[1]Computed by OEChem 2.3.0 (PubChem release 2025.04.14).

Table S4. Physicochemical and pharmacokinetic properties of citronellol (CIt), 1,8-cineole (CIn), limonene (LI), eugenol (EU), α-pinene (α-P), and carvone (CV) computed from the chemical structure and the canonical smiles, obtained from the PubChem database [42] and calculated with the web tools SwissADME [56] and pkCSM-pharmacokinetics [57].

Computed property	CIt	CIn	LM	EU	α-P	CV
Molecular Weight (MW, g/mol)[a]	156.26	154.25	136.23	164.20	136,23	150.22
XLogP3-AA - Lipophilicity[a]	3.2	2.5	3.4	2	2.80	2.40
Hydrogen Bond Donor Count (HBDC)[a]	1	0	0	1	0	0
Hydrogen Bond Acceptor Count (HBAC)[a]	1	1	0	2	0	1
Rotatable Bond Count (RBC)[a]	5	0	1	3	0	1
Topological Polar Surface Area (TPSA, Å^2)[a]	20.2	9.2	0	29.5	0.00	17.10
Complexity[a]	112	164	163	145	186	223
Vapor pressure (mmHg at 25 °C)[a]	0.04	1.9	1.55	0.01	4.75	0.16
Consensus Log $P_{o/w}$ - Lipophilicity[b]	2.92	2.67	3.37	2.25	3.44	2.44
[1]Log S - Water Solubility[b]	-3.06	-2.52	-3.35	-2.59	-3.31	-2.43
[2]Blood brain barrier (BBB) permeant[b]	Yes	Yes	Yes	Yes	Yes	Yes
Log Kp - Skin permeation (cm/s)[b]	-4.48	-5.30	-3.89	-5.69	-3.95	-5.29
Human Intestinal absorption (HIA, %)[c]	92.83	96.505	95.898	92.041	96.04	97.70
BBB permeability – Distribution (log BB)[c]	0.627	0.368	0.732	0.374	0.79	0.59
CNS permeability – Distribution (log PS)[c]	-2.222	-2.972	-2.37	-2.007	-2.01	-2.48

[a]PubChem [41]; [b]SwissADME [56]; [c]pkCSM-pharmacokinetics [56]; [1]Media of Log S (ESOL), Log S (Ali), and Log S (SILICOS-IT) [56]. [2]BOILED-Egg (yolk) [57].
